# Effects of selection for fast growth on survival rate during grow-out phase in giant freshwater prawn (*Macrobrachium rosenbergii*)

**DOI:** 10.1186/s12863-017-0521-7

**Published:** 2017-06-21

**Authors:** Nguyen Thanh Vu, Trinh Quoc Trong, Nguyen Hong Nguyen

**Affiliations:** 1 National Breeding Centre for Southern Freshwater Aquaculture, Research Institute for Aquaculture No. 2, 116 Nguyen Dinh Chieu Street, District 1, HCM City, Vietnam; 2 Faculty of Science, Health, Education and Engineering, University of the Sunshine Coast, Maroochydore, QLD 4558 Australia

**Keywords:** Survival rate, Correlated genetic changes, Selection response, Heritability and prawn

## Abstract

**Background:**

Correlated genetic response in survival to selection for high growth has not been reported in giant freshwater prawn (GFP) (*Macrobrachium rosenbergii*). The main aim of this study was to measure genetic changes and estimate heritability for this character (survival rate) and its genetic associations with body traits in a GFP population selected over eight generations from 2008 to 2015. Statistical analyses were conducted on 106,696 data records, using threshold logistic mixed model.

**Results:**

The estimated heritability for survival was 0.14 ± 0.04 and significant. Genetic associations of survival with body traits (weight, length and width) were weak, with the estimates of genetic correlations between the traits close to zero. Realised genetic changes in survival, calculated as the difference in estimated breeding values between the selection line and control group within the same generation, was in positive direction but the estimates were not significantly different from zero regardless of the expression unit used either in actual unit of measurement or genetic standard deviation unit. On the other hand, communal testing of stocks in the latest generation, namely G7 (2015), showed that the selection line had 18% higher survival rate than progeny of the wild prawns originated from Mekong river. This result suggests that inadvertent changes in survival occurred during domestication-selection.

**Conclusions:**

It is concluded that selection for high growth had no significant effect on survival in the present population of *M. rosenbergii.*

## Background

In commercial aquaculture sector, productivity or production yield is simply defined as a function of body weight and survival rate (yield = body mass × % survival). In addition to body weight, survival is one of the main characters to determine profit and economic return of the sector because it is related to the number of animals to be harvested and marketed.

Majority of selective breeding programs in aquaculture species have focused mutually exclusive on improving body weight or growth [1, 2]. Genetic evaluation of these selective breeding programs in fish showed that selection for high growth did not have significant effects on survival during grow-out, such as in tilapia [3] or common carp [4]. In some studies, improving growth performance was found to be associated with inadvertent increase in survival rate, an example can be found here in yellowtail kingfish [5]. To the best of our knowledge, there is no formal publication on correlated genetic changes in survival to selection for high growth in any shrimp species. Only the study of Campos-Montes et al. [6] reported a moderate and positive genetic correlation between body weight and survival in Pacific white leg shrimp.

Family selection programs have been initiated for giant freshwater prawn *M. rosenbergii* in China [7], India [8] and Vietnam [9, 10]. Selection for high growth significantly increased harvest body weight at an average rate of about 7% per generation [11]. There were also correlated changes in carcass traits (i.e. abdominal weight, telson-off weight and skeleton-off weight) by 3–4% per generation [12]. However, evaluation of possible changes in survival has not been conducted in this GFP population. To fill in the knowledge gap identified from the literature, in the present study we measured correlated response in this trait from a family selection program for GFP conducted over eight generations from 2008 to 2015. We also report heritability that estimated for survival and its genetic associations with important body traits (live weight, total length and abdominal width) as well as survival rate of the selected line in comparison with one of the founder stocks used to form the base population.

## Methods

### Experimental location and animals

The experiment was carried out at National Breeding Centre for Southern Freshwater Aquaculture (NABRECSOFA) at Cai Be district, Tien Giang province (latitude: 10.3 and longitude: 105.9), Vietnam. NABRECSOFA is one of the research centres of Research Institute for Aquaculture No. 2 (RIA2).

The present GFP population was established in 2007, comprising two indigenous wild strains (collected from Mekong and Dong Nai rivers that are geographically isolated) and an introduced strain from Malaysia. Details of the diallel cross to form the base population were described by Hung et al. [10]. In brief, the complete diallel cross involved three strains and produced 81 full-sibs families in 2008 (G0). From G1 – G7 (2009–2015), superior (highest ranking EBV, estimated breeding values) animals were selected to produce subsequence generations. In the last two generations, some males from Dong Nai and Mekong strains were mated with selected females due to high mortality of male brooders. In generation 2012 (G4), two exotic stocks imported from Thailand and Myanmar were introduced into the population at a ratio of 10% of selected breeders to produce G4.

### Family production, breeding, rearing and grow-out

In all generations (G0 – G7), family production, breeding, rearing and grow-out were practised as described in Hung et al. [10]. Indoor fibre glass tanks (1 cubic meter) were used for stocking selected broodstocks and mating. To produce half-sib families, 6–8 females were mated with one male, berried females were removed every 10 days after mating. Hatching of each family occurred in separate spawning tanks which was the same as larvae stocking stage. Rearing of prawn larvae were conducted in 70 l-round plastic tank with a density of 60 larvae per litre. After 25–30 days of rearing, post-larvae (PL) were stocked in 1 m^3^ tanks (at a density of 500–1000 PLs) for about 2 weeks until PL prawns were bigger than the mesh size. After a rearing period of about 2.0–2.5 months in hapas (net enclosure) installed in earthen pond where the PLs reached a size of larger than 1 g in body weight, 100–150 animals randomly sampled from each family were tagged using Visible Implant Elastomer [13].

All the tagged animals were then conditioned in tanks for two days without food before they were transferred to ponds for grow-out. In the grow-out phase, the stocking densities in every generation were from two to seven individuals/m^2^ with earthen pond sizes ranging from 2.000 to 9.000 m^2^. Industrial feed pellet (30% crude protein and 5% of fat) for black tiger shrimp (*P. monodon*) was used in the experiments with a diet of 3–5% biomass stocking rate [14]. We harvested the animals after 3–4 months of grow-out.

### Measurements and data collection

A detailed description of data recording for body traits (body weight, total body length, cephalothorax length, abdominal length and abdominal width), male prawn morphotypes and female reproduction status were given in Hung et al. [11].

In the present study, survival rate was recorded by the end of communal growing period, that is, at the time of harvest. The tag loss across generations were around 1% and animals that did not recognize family were assumed dead. Survival rate was recorded as a binary trait, coded as 1 for shrimp that survived at harvest or 0 for those that were not present or missing.

### Statistical analysis

#### Co-variance component analysis

Survival rate during grow-out was analysed using generalised linear mixed model (GLMM) with a logit link function. Under this model, the assumption was that the data followed a binomial distribution, and a logit link function $\left(\widehat{\mathrm{p}}\kern0.5em ={\mathrm{e}}^{\mathrm{x}}/\left({1+\mathrm{e}}^{\mathrm{x}}\right)\right)$ was used where *p* is the probability of animal survival recorded at harvest and *x* is a linear predictor. The fixed effects (***F***) included in the model were generation (spawning seasons, 2008 to 2015), line (selection or control) and pond (two grow-out ponds used per generation), the second order interaction between generation and line, and a linear covariate (i.e. number of days or age from stock to harvest) fitted within ponds and generation subclasses. The threshold logistic mixed model included individual prawn (***a***) as the random term effect [Eq. 1]. The mathematical form of the nonlinear (generalised) mixed models used to estimate variance components for survival rate is as the followings:

1 $$ \log \left(\frac{p_{ijk}}{1-{p}_{ijk}}\right)\kern0.5em =\mu +\mathbf{\mathsf{F}}+\mathbf{\mathsf{a}}+\mathbf{\mathsf{e}} $$

With the GLMM model [1], heritability was calculated using the variance of the logit link function, which implies a correction of the residual variance by factor π^2^/3.

$$ {h}^2=\frac{4{\sigma}_a^2}{\sigma_a^2+{\sigma}_e^2\frac{\pi^2}{3}} $$

where ${\sigma}_a^2$is the additive genetic variance and ${\sigma}_e^2=1$

The estimates of *h* ^*2*^ on the liability scales (logit) for binomial characters can be transformed to observed (0/1) scale using the formula of Robertson and Lerner [15] as follows:

$$ {h}_O^2={h}_L^2\frac{z^2}{p\left(1- p\right)} $$

where ${h}_O^2$ is the heritability on the observed (0/1) scale, ${h}_L^2$ is the estimated heritability on the liability (logit or probit) scale, *p* is a proportion of survival in the data, and *z* is the height of the ordinate of normal distribution corresponding to a truncation point applied to *p* proportion of survival during grow-out phase.

In addition to the GLMM procedure, the linear mixed model was also used to analyse body traits (weight, length and width). The fixed effects (***F***) were the same as described above in Eq. 1 but the random factors were the additive genetic effect of individual animal (***a***) and the common full-sib effect (***c***) [Eq. 2].

2 $$ y=\mu +\boldsymbol{F}+\boldsymbol{a}+\boldsymbol{c}+\boldsymbol{e} $$

Genetic and phenotypic correlations between survival and body traits were estimated using a series of two- trait linear mixed model [Eq. 2]. All computation was conducted in ASReml 3.0 [16]. Significance of the heritability and genetic (phenotypic) correlation estimates was tested using z-score against a large random normal distribution [17].

#### Selection response

Correlated genetic changes in survival rate were calculated using two methods: i) as the differences in estimated breeding values (EBVs) between the selection line and control group within the same generation, and ii) as the differences in the EBVs between successive generations. The responses for the trait studied were expressed in actual units (i.e. % for survival), genetic standard deviation unit (SD_A_) and percentage of the base population. The statistical model used to estimate EBVs for body weight and other traits were the same as those used to estimate the heritability.

## Results

### Pedigree information, data structure and basic statistics

Survival rate during grow-out of this giant freshwater prawn population (GFP) ranged from 20 to 44%, averaging 32.5% across generations (Table 1). One exception was generation 2014 (G6) where mass mortality occurred due to testing of different tagging methods. The survival rate in this generation was only about 15%.

**Table 1:** Number of sires, dams and offspring and survival rate over eight generations of selection

Generation	Line	Sires	Dams	Progeny	Survival rate (%)
G0 (2008)	Base population	81	81	1870	26.7
G1 (2009)	Selection Control	76 17	89 17	3060 711	40.2 32.4
G2 (2010)	Selection Control	60 20	96 20	4464 1256	45.4 40.4
G3 (2011)	Selection Control	65 42	144 42	5520 1324	31.3 38.5
G4 (2012)	Selection Control	74 27	93 34	2306 734	35.1 24.9
G5 (2013)	Selection	60	60	5743	33.8
G6 (2014)	Selection Control	34 7	54 15	504 162	20.4 10.0
G7 (2015)	Selection Control	56 25	87 31	3862 788	44.4 31.6

**Fig. 1:**
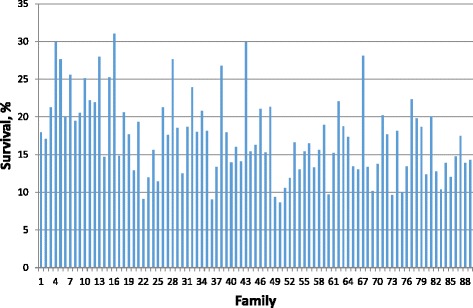
Variation in survival rate among 88 families in the latest generation 2015

In all generations, there was significant variation in survival rate among families (*P* < 0.001). An example of family variation in the latest generation 2015 (G7) is graphically presented in Fig. 1. Analysis of variance showed that survival rate differed significantly among 88 families in this generation. For instance, the highest survival family was 31%, whereas the poorest survival family was only 8.6%.

### Heritability

The heritability estimated from logistic generalised model for survival rate during grow-out was 0.14 ± 0.04 and differed significantly from zero (Table 2). When the estimate on the underlying liability was transformed to the observed scale (0, 1), it had similar magnitude to the heritability obtained from threshold model.

**Table 2:** Heritability and common full-sib effects for survival and body traits

Traits	Model	Heritability	Full-sib effects
Survival	1	0.14 ± 0.04	
Weight	2	0.12 ± 0.02	0.07 ± 0.01
Length	2	0.09 ± 0.02	0.09 ± 0.01
Width	2	0.21 ± 0.03	0.04 ± 0.01

Model 1 = Threshold logistic model, Model 2 = The animal model with common full-sib effect

Further, the heritability estimate obtained for body traits (weight, length and width) from linear mixed model analysis was generally moderate, ranging from 0.09 to 0.21. The proportion of maternal and common environmental effects accounted for 4 to 9% of total phenotypic variation in morphometric body traits (Table 2).

### Phenotypic and genetic correlations

Table 3 presents phenotypic and genetic correlations between survival and body traits. All the estimates of genetic and phenotypic correlations were low and not significantly different from zero (*P* > 0.05).

**Table 3:** Phenotypic (r_p_) and genetic (r_g_) correlations between survival rate and body traits

Traits	r_p_	r_g_
Weight	−0.01 ± 0.007	−0.10 ± 0.09
Length	−0.01 ± 0.006	−0.04 ± 0.09
Width	−0.02 ± 0.006	−0.10 ± 0.09

### Correlated genetic response in survival

Correlated genetic changes in survival to selection for high growth measured as the differences in EBV between the selection line and control (method 1) and between successive generations (method 2) are shown in Table 4. Consistently across the two methods, the estimated correlated responses in survival were close to zero and not significant, regardless of the expression units used either in actual unit of measurements or genetic standard deviation unit (i.e. 0.009 to 0.046SD_A_). When the response in survival was expressed as percentage of the population mean, it ranged from 0.03 to 0.13% across generations of selection.

**Table 4:** Correlated genetic changes in survival rate measured as the differences in EBV between the selection line and control group (method 1) or between successive generations (method 2)

Generation	Method 1			Method 2		
	Actual unit	SD_A_	% of the mean	Actual unit	SD_A_	% of the mean
1	0.00037					
2	0.000119	0.02927	0.07827	0.000405	0.000000	0.00000
3	0.000267	0.00942	0.02518	0.000634	0.031983	0.085516
4	0.000492	0.02113	0.05650	0.000614	0.050087	0.133924
5	0.000395	0.03890	0.10400	0.000466	0.048517	0.129725
6	0.00091	0.03123	0.08350	0.001671	0.036872	0.098589
7	n.a.	n.a.	n.a.	n.a.	n.a.	n.a.
8	0.00540	0.00000	0.00000	0.002915	0.00000	0.000000

Actual unit (%), genetic standard deviation unit (SD_A_), n.a. = not available due to limited data records

### Survival of the selected line compared with wild stocks

**Fig. 2:**
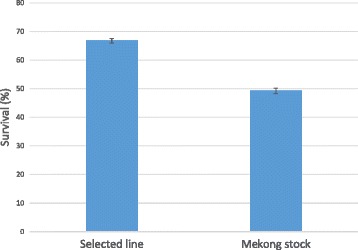
Survival of the selected line compared with the wild stock from Mekong river

Communal testing of the selected line together with its counterparts from the wild was conducted in ponds in 2015. Least squares means obtained from generalised logistic mixed model show that the selected line had significantly higher survival rate than the stock from Mekong river (*P* < 0.05) (Fig. 2).

## Discussion

The main finding of our study was that the selection program for high growth did not result in adverse effects on survival of GFP during grow-out phase. The correlated responses in survival, although in positive direction, were small and not significant. The negligible changes in survival during the course of eight generations of selection for high growth in this population of GFP are in accordance with the results reported in the literature from long term genetic improvement programs for other aquaculture species, such as tilapia [3, 18, 19], common carp [4] or salmonids [1]. Our present results taken collectively with those reported in other species suggest that improvement in survival rate by selection on body traits could be achieved but at a slow rate.

The small changes in survival here are consistent with the weak genetic associations between survival and body traits obtained in the present study, with the genetic correlation estimate between the two traits close to zero (Table 3). A similar trend was reported in fish and crustacean species, such as rainbow trout [20], tilapia [3], common carp [4] or Pacific white leg shrimp [6]. There are however estimates of the genetic correlations between the two traits which are moderate and either positive [21, 22] or negative [23].

In this study, the analysis of variance using threshold logistic mixed model showed that there was additive genetic component for survival during grow-out phase in ponds (h^2^ = 0.14 ± 0.04). This suggests that genetic improvement through direct selection for this trait (survival rate) should be possible. In a different population of GFP, Luan et al. [7] reported a low heritability for this character. Studies in other species pointed out that survivals in the early phase of development or during grow-out are lowly heritable such as in shrimp [6], freshwater fish (tilapia and common carp) [3, 4] or abalone [22]. On the other hand, a number of studies also reported moderate heritability for survival under field grow-out conditions for Atlantic salmon [24] or another population of common carp [25] and tilapia [18].

Despite the existence of the heritable additive genetic variation for survival in the present population of GFP, it is well known that this trait is strongly influenced by environmental factors. Among factors recorded in the present study, the effect of generations, grow-out pond, sex/morphotype on survival were all statistically significant (*P* < 0.05 to 0.001). Several other factors such as diseases or inbreeding may have had impacts on this trait; however, they were not examined in the present research. Our results also pointed out that there is a need to maintain the control group since it enabled the separation of the domestication-selection effect. Owning to the non-significant difference between the selection line and control group in this study, any increase in the survival rate in the selected line relative to its counterparts from the wild was likely a result from long-term domestication. The higher survival rate in the selected line compared with the wild stock would bring about greater economic benefits and returns to prawn farmers.

## Conclusion

- Selection for high growth did not cause significant changes in survival rate during grow-out phase in the present population of GFP
- The genetic correlations between survival and body traits were weak and not significant
- There is heritable additive genetic component for survival during grow-out
- Survival during grow-out was largely influenced by environmental factors
- Possible effect of selection for high growth on survival rate during the early phase of rearing in this GFP population deserves future research.

## References

[1] Gjedrem T (2010). The first family-based breeding program in aquaculture. Rev Aquac.

[2] Nguyen HN (2016). Genetic improvement for important farmed aquaculture species with a reference to carp, tilapia and prawns in Asia: achievements, lessons and challenges. Fish Fish.

[3] Hamzah A, Mekkawy W, Khaw HL, Nguyen NH, Yee HY, Abu Bakar KR, et al. Genetic parameters for survival during the grow-out period in the GIFT strain of Nile tilapia (*Oreochromis niloticus*) and correlated response to selection for harvest weight. Aquac Res. 2015; doi:10.1111/are.12859.

[4] Dong Z, Nguyen NH, Zhu W (2015). Genetic evaluation of a selective breeding program for common carp *Cyprinus carpio* conducted from 2004 to 2014. BMC Genet.

[5] Knibb W, Miller A, Quinn J, D'Antignana T, Nguyen NH (2016). Comparison of lines shows selection response in kingfish (*Seriola lalandi*). Aquaculture.

[6] Campos-Montes GR, Montaldo HH, Martínez-Ortega A, Jiménez AM, Castillo-Juárez H (2013). Genetic parameters for growth and survival traits in Pacific white shrimp *Penaeus (litopenaeus) vannamei* from a nucleus population undergoing a two-stage selection program. Aquac Int.

[7] Luan S, Yang G, Wang J, Luo K, Chen X, Gao Q, et al. Selection responses in survival of *Macrobrachium rosenbergii* after performing five generations of multi-trait selection for growth and survival. Aquac Int. 2014;22(3):993–1007.

[8] Pillai BR, Mahapatra KD, Ponzoni RW, Sahoo L, Lalrinsanga PL, Mekkawy W, et al. Survival, male morphotypes, female and male proportion, female reproductive status and tag loss in crosses among three populations of freshwater prawn *Macrobrachium rosenbergii* (de Man) in India. Aquac Res. 2015;46(11):2644–55.

[9] Thanh NM, Nguyen NH, Ponzoni RW, Vu NT, Barnes AC, Mather PB (2010). Estimates of strain additive and non-additive genetic effects for growth traits in a diallel cross of three strains of giant freshwater prawn (*Macrobrachium rosenbergii*) in Vietnam. Aquaculture.

[10] Hung D, Nguyen NH, Ponzoni RW, Hurwood DA, Mather PB: Quantitative genetic parameter estimates for body and carcass traits in a cultured stock of giant freshwater prawn (*Macrobrachium rosenbergii*) selected for harvest weight in Vietnam. Aquaculture 2013, 404–405(0):122-129.

[11] Hung D, Vu NT, Nguyen NH, Ponzoni RW, Hurwood DA, Mather PB: Genetic response to combined family selection for improved mean harvest weight in giant freshwater prawn (*Macrobrachium rosenbergii*) in Vietnam. Aquaculture 2013, 412–413(0):70-73.

[12] Hung D, Nguyen NH (2014). Modeling meat yield based on measurements of body traits in genetically improved giant freshwater prawn (GFP) *Macrobrachium rosenbergii*. Aquac Int.

[13] Hung D, Nguyen NH (2015). Are female reproductive status and male morphotypes of the giant freshwater prawn *Macrobrachium rosenbergii* altered by selection for high growth?. Mar Freshw Behav Physiol.

[14] Hung D, Nguyen HN (2014). Genetic inheritance of female and male morphotypes in giant freshwater prawn *Macrobrachium rosenbergii*. PLoS One.

[15] Robertson A, Lerner IM (1949). The heritability of all-or-none traits; viability of poultry. Genetics.

[16] Gilmour AR, Gogel BJ, Cullis BR, Welham SJ, Thompson R (2009). ASReml User Guide Release 3.0.

[17] Nguyen NH, Khaw HL, Ponzoni RW, Hamzah A, Kamaruzzaman N: Can sexual dimorphism and body shape be altered in Nile tilapia (*Oreochromis niloticus*) by genetic means? Aquaculture 2007, 272, Supplement **1**(0):S38-S46.

[18] Ninh NH, Thoa NP, Knibb W, Nguyen NH. Selection for enhanced growth performance of Nile tilapia (*Oreochromis niloticus*) in brackish water (15 - 20 ppt) in Vietnam. Aquaculture. 2014;428-429:1–6.

[19] Thoa NP, Ninh NH, Knibb W, Nguyen NH (2016). Does selection in a challenging environment produce Nile tilapia genotypes that can thrive in a range of production systems?. Sci Rep.

[20] Vehviläinen H, Kause A, Kuukka-Anttila H, Koskinen H, Paananen T (2012). Untangling the positive genetic correlation between rainbow trout growth and survival. Evol Appl.

[21] Thoa NP, Knibb W, Ninh NH, Van Dai N, Nhat PH, Toan LM, et al. Genetic variation in survival of tilapia (*Oreochromis niloticus*, Linnaeus, 1758) fry during the early phase of rearing in brackish water environment (5–10 ppt). Aquaculture. 2015;442:112–8.

[22] Liu J, Lai Z, Fu X, Wu Y, Bao X, Hu Z, et al. Genetic parameters and selection responses for growth and survival of the small abalone Haliotis diversicolor after four generations of successive selection. Aquaculture. 2015;436(0):58–64.

[23] Rye M, Lillevik KM, Gjerde B (1990). Survival in early life of Atlantic salmon and rainbow trout: estimates of heritabilities and genetic correlations. Aquaculture.

[24] Lillehammer M, Ødegård J, Madsen P, Gjerde B, Refstie T, Rye M (2013). Survival, growth and sexual maturation in Atlantic salmon exposed to infectious pancreatic necrosis: a multi-variate mixture model approach. Genet Select Evol.

[25] Nielsen HM, Ødegård J, Olesen I, Gjerde B, Ardo L, Jeney G, et al. Genetic analysis of common carp (*Cyprinus carpio*) strains: I: Genetic parameters and heterosis for growth traits and survival. Aquaculture. 2010;304(1):14–21.

